# PCDH8 is a novel prognostic biomarker in thyroid cancer and promotes cell proliferation and viability

**DOI:** 10.1007/s10142-024-01312-3

**Published:** 2024-02-17

**Authors:** Ruida Yang, Nan Yang, Pan Yin, Zihan Xue, Feidi Sun, Ruihan Fan, JiaFu Liang, Xinru Lv, Shaobo Wu, Liankang Sun

**Affiliations:** 1 Department of Hepatobiliary Surgery, The First Affiliated Hospital of Xi’an Jiaotong University, Xi’an, 710061 People’s Republic of China; 2 Department of Oncology, the First Affiliated Hospital of Xi’an Jiaotong University, Xi’an, 710061 People’s Republic of China

**Keywords:** PCDH8, Thyroid cancer, Prognosis, Cell proliferation, Biomarker, Clinical-pathological features

## Abstract

Protocadherin 8 (PCDH8), a calcium-dependent transmembrane protein in the protocadherin family, regulates cell adhesion and signal transduction. While some studies have provided indirect evidence that PCDH8 has cancer-promoting properties, this association is controversial. In particular, its involvement in thyroid cancer (THCA) remains unclear. We aimed to elucidate the role of PCDH8 in THCA using bioinformatic analysis. Subsequently, the results were experimentally validated. The analysis conducted using the R programming language and online web tools explored PCDH8 expression levels, prognostic, and clinical implications, and its relationship with the tumor immune microenvironment in THCA. Furthermore, we examined the association between PCDH8 and co-expressed genes, highlighting their involvement in several biological processes relevant to THCA. The potential of PCDH8 as a therapeutic target for this pathology was also explored. Immunohistochemical (IHC) staining was performed on samples from 98 patients with THCA, and experimental validation was carried out. PCDH8 was significantly elevated in cancer tissues and associated with poor prognosis, several clinical factors, and immune cell and checkpoint abundance. Cox regression and survival analyses, together with Receiver Operating Curves (ROC) indicated that PCDH8 was an independent prognostic factor for THCA. Furthermore, PCDH8 impacts cell viability and proliferation, promoting tumorigenesis. Also, it influences tumor cell sensitivity to various drugs. Thus, PCDH8 might be a potential therapeutic target for THCA. IHC, cell culture, MTT, and colony formation experiments further confirmed our findings. This analysis provided insights into the potential carcinogenic role of PCDH8 in THCA, as it impacts cell viability and proliferation. Thus, PCDH8 might play an important role in its prognosis, immune infiltration, and diagnosis.

**Supplementary Information:**

The online version contains supplementary material available at 10.1007/s10142-024-01312-3.

## Introduction

The absence of protocadherin (PCDH) proteins, such as PCDH8, impacts the development of various types of human cancers, including colon, liver, renal, prostate, breast, nasopharyngeal, and lung cancer, as well as astrocytoma (Stassar et al. 2001; Chen et al. 2002; Okazaki et al. 2002; Waha et al. 2005; Imoto et al. 2006; Ying et al. 2006; Yu et al. 2008, 2009). This absence can be attributed to gene deletion, mutation, or promoter methylation (Waha et al. 2005; Imoto et al. 2006; Ying et al. 2006). Conversely, PCDH overexpression impedes anchorage-dependent and anchorage-independent tumor cell proliferation and migration (Waha et al. 2005; Imoto et al. 2006; Ying et al. 2006). However, conflicting evidence exists regarding the involvement of PCDH8 in cancer. One study revealed that microRNA-429 upregulates PCDH8, increasing the migratory capacity of endometrial carcinoma cells through the enhancement of epithelial-mesenchymal transition (EMT) (Yu et al. 2009). However, a debate persists regarding its overall impact.

Thyroid cancer (THCA), the most common endocrine cancer worldwide is typically treated with conventional modalities such as major surgery, thyroid stimulation, hormone suppression, and 131I therapy which have shown satisfactory efficacy (Verburg et al. 2017). Despite a high five-year survival rate exceeding 97% and a ten-year rate of 85%, 10 to 28% of patients experience relapse 10 to 27 years after treatment (Grogan et al. 2013; Schneider and Chen 2013; Riesco-Eizaguirre et al. 2016; Luo et al. 2019a, b). However, the incidence and mortality of THCA have gradually increased in the last decade (Sturgeon et al. 2016). Moreover, THCA is a chronic disease, necessitating long-term healthcare and medication (Jiang et al. 2019). However, for aggressive malignant forms, 5–20% of patients who undergo total thyroidectomy experience local recurrence or distant metastasis (Pellegriti et al. 2004; American Thyroid Association Guidelines Taskforce on Thyroid et al. 2009). Novel biomarkers are needed to enhance understanding and prevention of the disease and provide better follow-up diagnosis, leading to a tailored treatment. PCDH8, with its unique characteristics and functions, may be associated with THCA, though the relationship remains unclear. In our study, we analyzed the Cancer Genome Atlas (TCGA) database to investigate the differential expression of PCDH8 between paired paracancerous thyroid tissues and tumor tissues and used human THCA samples to validate our analysis. We employed enrichment analysis techniques to explore potential mechanisms underlying the involvement of PCDH8 in THCA tumorigenesis. Cox regression and survival analyses were performed to assess its diagnostic and prognostic value.

We investigated the role of PCDH8 in immune infiltration and its functions in the tumor microenvironment. Additionally, we explored the targets of PCDH8-related kinases, studied drug sensitivities, and provided potential insights for future interventions in THCA treatment. Combining immunohistochemistry (IHC), cell culture, 3-(4,5-dimethylthiazol-2-yl)-2,5-diphenyl-2H-tetrazolium bromide (MTT), and colony formation assays, we concluded that PCDH8 is an important prognostic biomarker involved in tumorigenesis through the regulation of cell proliferation and activity.

## Materials and methods

### Public cohort

We extracted THCA mRNA expression data (FPKM) and clinical information from the Cancer Genome Atlas (TCGA) database (https://portal.gdc.cancer.gov/), including 510 tumor and 58 normal samples. The expression matrix in FPKM format and clinical data are available as Supplementary materials (Supplement 1: RNA_seq_FPKM and Supplement 4: Clinical). Throughout the study, we adhered to TCGA’s publication guidelines for the appropriate data use and reporting.

### Human THCA samples and IHC

From January 2013 to December 2018, 98 human papillary thyroid carcinoma samples and their adjacent non-tumorous specimens were collected at the First Affiliated Hospital of Xi’an Jiaotong University. IHC staining was performed to examine the expression of PCDH8 as previously described (Sun et al. 2020). The anti-PCDH8 antibody was purchased from Santa Cruz Biotechnology (#sc-377348) and was utilized at a working concentration of 1:50. All samples underwent histopathological validation, confirming their diagnostic accuracy. Patients associated with these samples had not undergone any radiotherapy, chemotherapy, or I^131^ treatment before surgery. The study adhered to the ethical standards of the Research Ethics Committee of The First Affiliated Hospital of Xi’an Jiaotong University and the 1964 Helsinki Declaration and its later amendments. Written informed consent was obtained from all patients included in this study. Baseline data for the 98 patients are provided as Supplement 5 Baseline of patients.

### PCDH8 expression in pan-cancer and THCA

We examined PCDH8 expression in 33 types of human cancer using THCA data from TCGA and the R package. We used gene set cancer analysis (GSCA) for visualization of pan-cancer analysis. PCDH8 expression levels were compared between THCA tissues and adjacent normal tissues using unpaired and paired t-tests. Differentially expressed genes (DEGs) were identified using the limma software package (Ritchie et al. 2015) with cutoffs of log|FC|> 1.0 and *p*-value < 0.05. Furthermore, we compared PCDH8 expression between normal and thyroid cancer tissues using the Human Protein Atlas database (HPA) (www.proteinatlas.org) (Ponten et al. 2008). The HPA database uses antibody profiling for precise protein localization, offering access to protein expression profiles in 32 human tissues.

### Genetic alterations and the mutation landscape of PCDH8 in THCA

We obtained RNA-sequencing profiles, genetic mutation data, and clinical information for THCA from the TCGA dataset. Mutation data were downloaded and visualized using the maftools package in R software. Details are available in the Supplementary information (Supplement 2).

### Clinical factor analysis

We compiled the clinical characteristics of patients with thyroid cancerTHCA and analyzed their correlation with PCDH8 expression level. Additionally, we explored the relationship between PCDH8 and certain clinical tumor markers using the ggplot2 package in R.

### Survival and prognosis analyses

The area under the curve (AUC) of receiver operating characteristic (ROC) curves was used to analyze the diagnostic value of PCDH8 in THCA. Utilizing Kaplan–Meier plots, hazard ratios (HR), and log-rank p-values, we analyzed the relationship between PCDH8 and survival in THCA.

For evaluating the association between progression-free interval (PFI) and clinical-pathological factors in THCA, we carried out both univariate and multivariate Cox regression analyses. ROC curves for diagnosis and nomogram were drawn using R packages (pROC and survival). All analyses were based on patients with tumors included in the public cohort. PFI is defined as the duration during which a patient survives without experiencing further deterioration of the disease after receiving treatment. The outcome indicator for PFI is based on the occurrence of either deterioration or death (Supplement 4 Clinical).

### Protein–protein (PPI) network, Gene Ontology (GO), and Kyoto Encyclopedia of Genes and Genomes (KEGG) enrichment analyses of DEGs

Utilizing transcriptome data of 568 patients from the TCGA-THCA database, we categorized them into high and low PCDH8 expression groups based on the median value. Differential analysis, using the limma package, identified 415 genes (Supplement 3 DEGs-logFC2). Subsequently, we carried out a GO (Gene Ontology 2006) analysis to explore specific biological properties, encompassing biological processes, cellular components, and molecular functions. KEGG enrichment analysis (Kanehisa et al. 2017) was carried out to identify pathways associated with tumor occurrence and development. Results from GO and KEGG analyses were processed using enrichplot, clusterProfiler, and ggplot2 packages. The PPI network of PCDH8 was established from the STRING database and visualized using GeneMANIA. A statistically significant difference and enrichment were defined as *p*-values < 0.05 and counts over 6, respectively.

### Gene set enrichment analysis (GSEA)

Furthermore, we categorized 568 THCA samples into high and low PCDH8 expression groups based on the median value. GSEA was utilized to highlight significant differences between the two expression groups and identify correlations between gene expression and biological pathways. The calculations were repeated 1000 times, considering results with *p*-values < 0.05 and *q*-values < 0.05 as statistically significant.

### Immune infiltration analysis

We examined the relationship between PCDH8 expression and immune responses in THCA, focusing on the abundance of tumor-infiltrating immune cells and relevant molecules. Furthermore, we evaluated the impact of PCDH8 on immune checkpoints in THCA utilizing the ggplot2 package in R and web tools. Additionally, immune infiltration levels were evaluated using algorithms such as EPIC and MCP-counter, and the tumor mutation burden (TMB) score was calculated. These analyses collectively offer comprehensive insights into the immune landscape of THCA and its potential implications in tumor development and immunotherapy response.

### LinkedOmics and drug sensitivity analysis

This study utilized LinkedOmics, a comprehensive platform for multi-omics data evaluation across various tumor types (Vasaikar et al. 2018). The LinkInterpreter module from LinkedOmics was used to investigate the PCDH8 kinase target. GSCALite (http://bioinfo.life.hust.edu.cn/web/GSCALite/), an online tool, facilitated gene cluster analysis in tumors (Liu et al. 2018). We analyzed the relationship between PCDH8 and drug sensitivity expressed as the inhibitory concentration 50 (IC50), using 481 drugs obtained from the Therapeutics Response Portal and applying Spearman correlation.

### Cell culture

Human thyroid carcinoma cell lines (TPC-1 and FTC-133) and thyroid epithelial cell line Nthy-ori3-1 were obtained from Cobioer Biosciences (Nanjing, Jiangsu, China). All cells were cultured in Roswell Park Memorial Institute-1640 medium (Gibco, Hangzhou, China) containing 10% fetal bovine serum (Gibco Invitrogen, Grand Island, NY, United States), 100 µg/mL streptomycin, and 100 U/mL penicillin.

### Assessment of PCDH8 knockdown efficiency in THCA cells

After transfecting PCDH8 shRNA into TPC-1 and FTC-133 cells for 48 h or 72 h, we collected mRNA and protein from these cells to validate the knockdown efficiency of PCDH8 in these cells using quantitative reverse transcription polymerase chain reaction (qRT-PCR) and Western blot, respectively. The utilized protocols have been previously described (Sun et al. 2020). The primers for qRT-PCR were: PCDH8 forward: CAGTCCGATACAGCACCTTC; PCDH8 reverse: GCTTGTGTCACCCGATACTT; GAPDH forward: GGTGTGAACCATGAGAAGTATGA; GAPDH reverse: GAGTCCTTCCACGATACCAAAG. Anti-PCDH8 and anti-GAPDH antibodies for Western blot were purchased from Santa Cruz Biotechnology (#sc-377348; 1:500) or Abcam (#ab8245; 1:2000) respectively.

### MTT and colony formation assay

The PCDH8 shRNA depletion (#sc-76085) was purchased from Santa Cruz Biotechnology, Inc. Following the designated intervention, THCA cells were used for assessing cell viability and proliferation using MTT assay and clone formation assay, respectively. The utilized protocols have been previously published (Sun et al. 2020).

### Statistical analysis

Statistical analysis was carried out using R and GraphPad Prism 5. For correlation analyses, we employed Spearman’s test. The results were considered significant for *p*-values < 0.05.

## Results

### Pan-cancer analysis and differential expression of PCDH8 in THCA

**Fig. 1:**
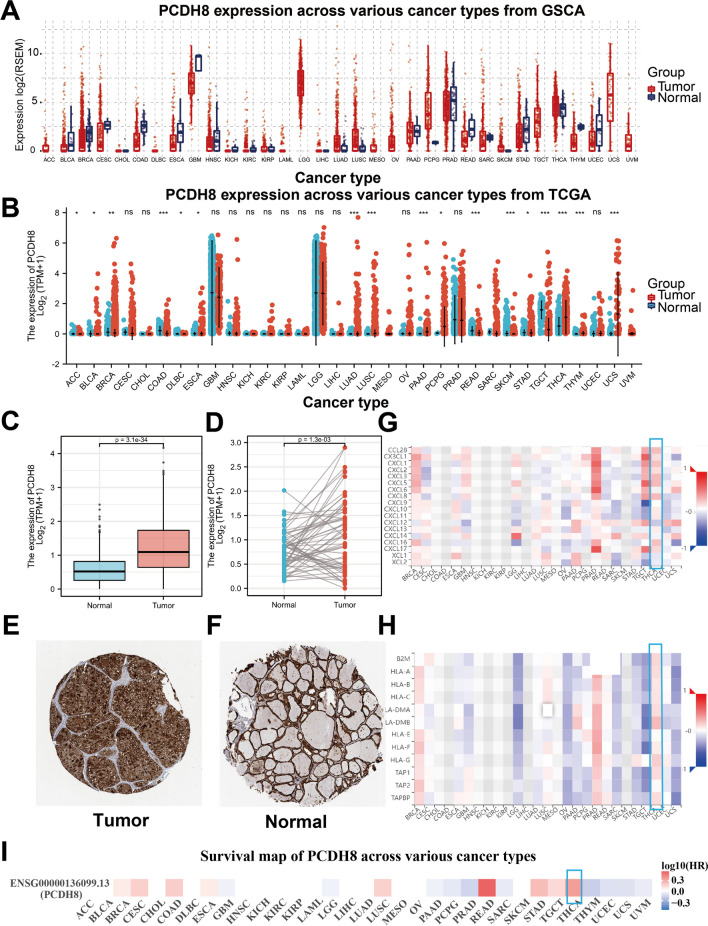
PCDH8 was differentially expressed in THCA and many other cancers. (**A**, **B**) The PCDH8 mRNA expression in tumor and normal thyroid tissues. (**C**, **D**) The PCDH8 mRNA expression in paired THCA samples and unpaired THCA samples. (**E**, **F**) The PCDH8 protein expression in normal thyroid and THCA tissues. (**G**, **H**, **I**) The PCDH8 pan-carcinoma-specific analysis of chemokines, MHC and Survival in THCA. *p < 0.05; **p < 0.01; ***p < 0.001

In our study, we initially assessed the expression of PCDH8 across 32 types of human cancer. As illustrated in Fig. 1A, B, PCDH8 expression varied among these cancer types compared to their corresponding normal tissues. Remarkably, higher expression of PCDH8 was observed in several cancers, such as bladder urothelial carcinoma, breast invasive carcinoma, colon adenocarcinoma, lung squamous cell carcinoma, pancreatic adenocarcinoma, pheochromocytoma, paraganglioma, THCA, esophageal carcinoma, thymoma, and uterine carcinosarcoma. However, PCDH8 expression did not change in cervical squamous cell carcinoma, endocervical adenocarcinoma, cholangiocarcinoma, kidney renal clear cell carcinoma, kidney renal papillary cell carcinoma, prostate adenocarcinoma, and uterine corpus endometrial carcinoma.

In terms of THCA, both paired and unpaired t-tests indicated that PCDH8 expression was significantly increased in tumor tissues (Fig. 1C, D). Based on the HPA database, their IHC results showed that the protein expression level of PCDH8 was significantly higher in thyroid cancer tissues than in normal tissues (Fig. 1E, F). Furthermore, we observed distinctive patterns and performance of PCDH8 in THCA, particularly concerning chemokines, major histocompatibility complex, and survival (Fig. 1G, H, I). These findings suggest that PCDH8 may have unique roles and specific implications in THCA, extending beyond its general expression in other cancers.

### Clinical, pathological, and prognosis analyses

**Fig. 2:**
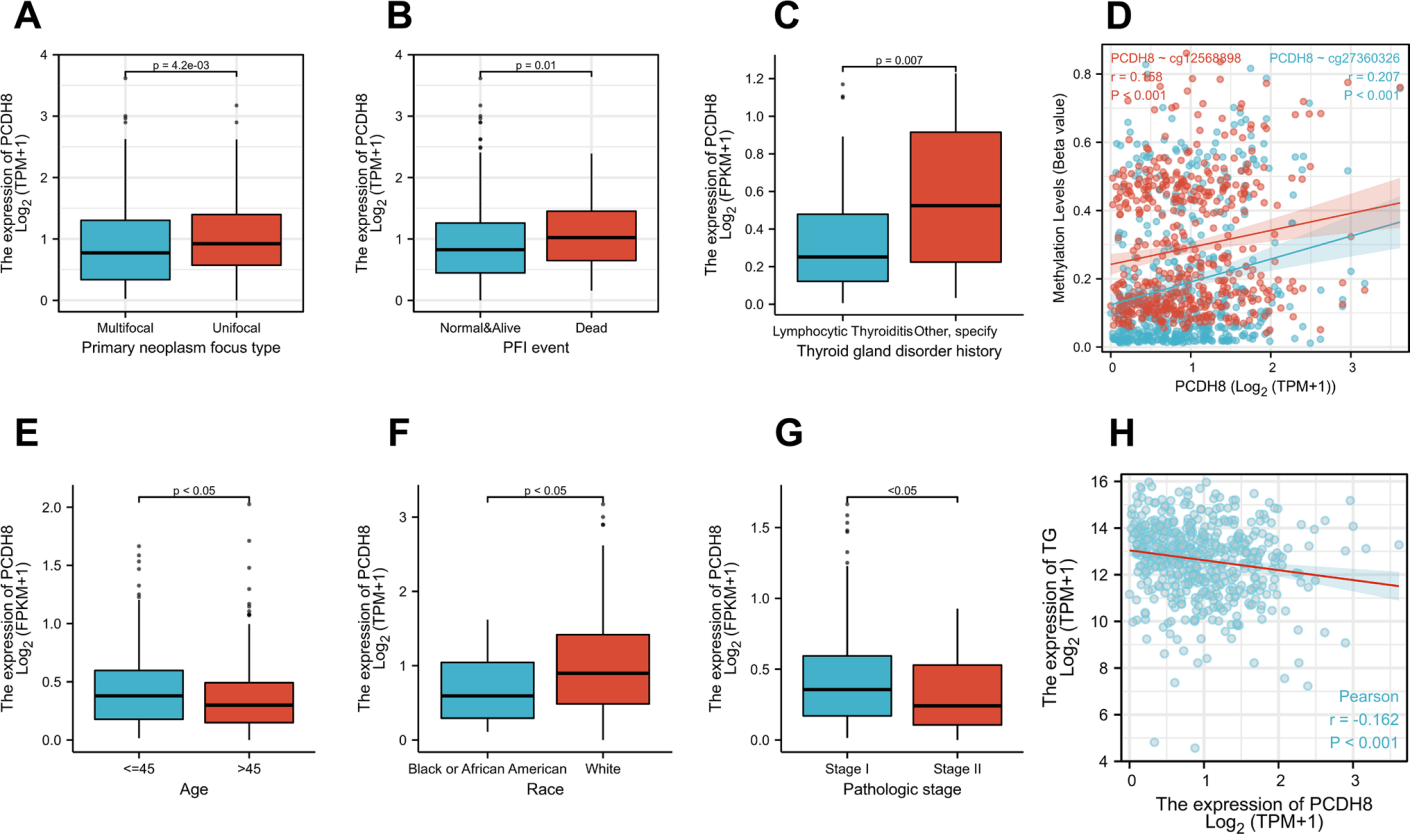
The clinical value of PCDH8 in THCA. (**A**) The association of PCDH8 expression with primary neoplasm focus type of THCA patients. (**B**) The association of PCDH8 expression with PFI of THCA patients. (**C**) The association of PCDH8 expression with thyroid gland disorder history of THCA patients. (**D**) The association of PCDH8 expression with Methylation levels of THCA patients. (**E**) The association of PCDH8 expression with age of THCA patients. (**F**) The association of PCDH8 expression with Race of THCA patients. (**G**) The association of PCDH8 expression with Pathologic stage of THCA patients. (**H**) The association of PCDH8 expression with TG (thyroglobulin) of THCA patients. PFI, progression free interval

We analyzed the correlation between PCDH8 and several key clinical factors in THCA. These findings are summarized in Table 1. Our results indicate that the expression of PCDH8 is significantly associated with the type of primary neoplasm focus (Table 1, Fig. 2A), the PFI (Fig. 2B), history of thyroid gland disorders (Fig. 2C), methylation levels (Fig. 2D), age (Fig. 2E), race (Fig. 2F), pathologic stage (Fig. 2G), and thyroglobulin levels (Fig. 2H). However, no significant correlation was observed between PCDH8 and overall survival (OS), possibly due to the generally favorable prognosis of thyroid cancer.

**Table 1:** Some representative clinical factors correlated with PCDH8 in THCA

Characteristic	Low expression of PCDH8	High expression of PCDH8	p
n	255	255	
T stage, n (%)			0.492
T1	75 (14.8%)	68 (13.4%)	
T2	87 (17.1%)	80 (15.7%)	
T3	82 (16.1%)	93 (18.3%)	
T4	9 (1.8%)	14 (2.8%)	
N stage, n (%)			0.455
N0	109 (23.7%)	120 (26.1%)	
N1	119 (25.9%)	112 (24.3%)	
M stage, n (%)			0.095
M0	150 (50.8%)	136 (46.1%)	
M1	2 (0.7%)	7 (2.4%)	
Gender, n (%)			0.842
Female	187 (36.7%)	184 (36.1%)	
Male	68 (13.3%)	71 (13.9%)	
Age, n (%)			0.003
< = 45	103 (20.2%)	138 (27.1%)	
> 45	152 (29.8%)	117 (22.9%)	
Primary neoplasm focus type, n (%)			0.016
Multifocal	129 (25.8%)	104 (20.8%)	
Unifocal	118 (23.6%)	149 (29.8%)	
Neoplasm location, n (%)			0.506
Bilateral	49 (9.7%)	39 (7.7%)	
Isthmus	12 (2.4%)	10 (2%)	
Left lobe	87 (17.3%)	90 (17.9%)	
Right lobe	101 (20%)	116 (23%)	
Thyroid gland disorder history, n (%)			0.029
Lymphocytic Thyroiditis	43 (9.5%)	31 (6.9%)	
Nodular Hyperplasia	40 (8.8%)	28 (6.2%)	
Normal	131 (29%)	154 (34.1%)	
Other, specify	8 (1.8%)	17 (3.8%)	
Age, median (IQR)	50 (36, 60)	42 (33, 57)	0.007

**Fig. 3:**
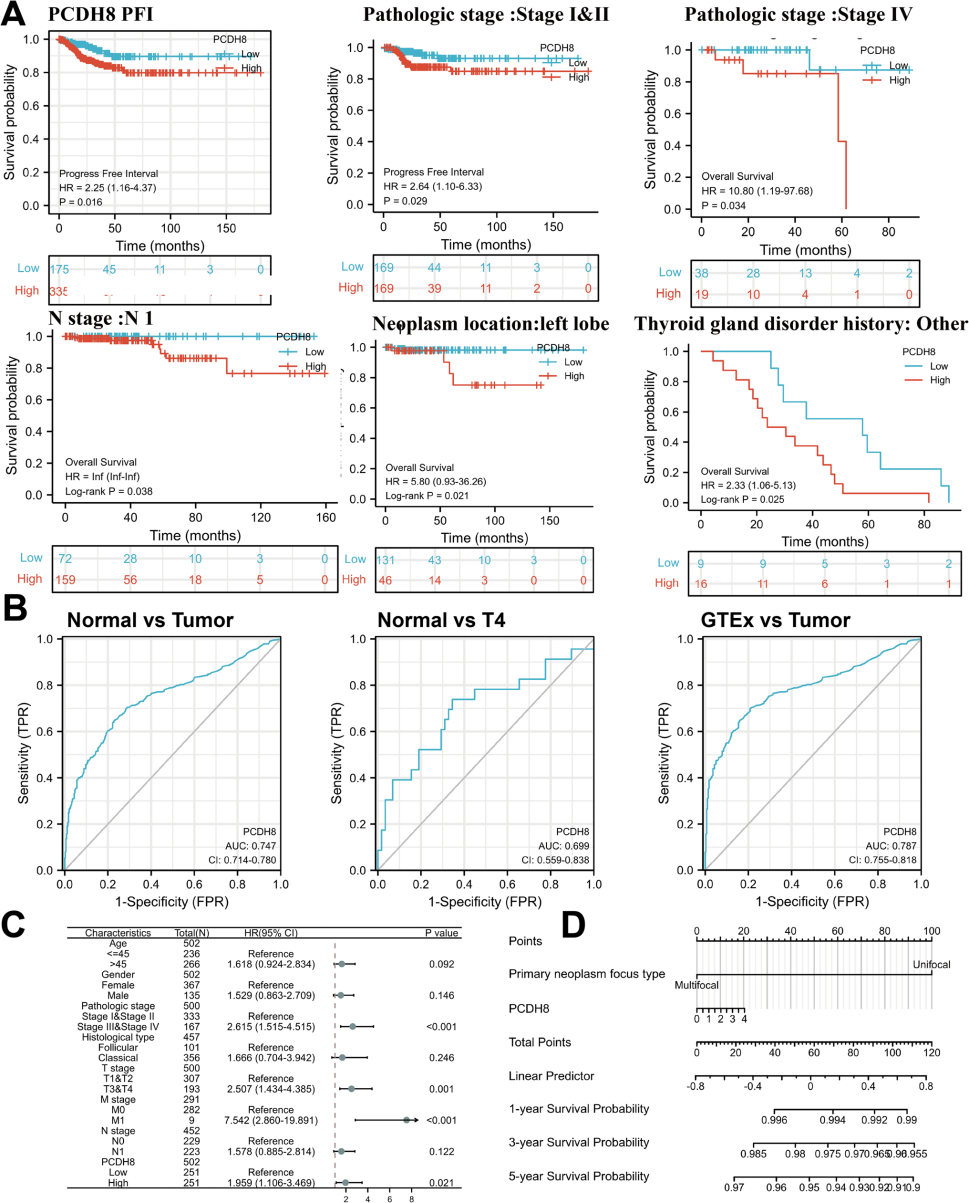
The prognostic value of PCDH8 in THCA. (**A**) The association of PCDH8 expression with PFI or OS of THCA patients or subgroup THCA patients using R. (**B**) The ROC curve of PCDH8 expression for predicting the survival status. (**C**, **D**) A nomogram model and forest diagram was created by combining clinical factors with the PCDH8 expression which illustrates the survival probabilities of patients at 1, 3, and 5 years. THCA, thyroid cancer; ROC, receiver operating characteristic; OS, overall survival; PFI, progress-free interval; HR, hazard ratio; AUC, area under the curve

Kaplan–Meier survival curve analysis revealed that patients with THCA and upregulated PCDH8 expression have a significantly poorer PFI than those with lower PCDH8 expression levels (*p* = 0.016, HR = 2.25 [1.16–4.37]) (Fig. 3A). Thus, an increased PCDH8 expression is associated with a worse prognosis in patients with THCA. Furthermore, our investigation revealed a strong correlation between PCDH8 overexpression and an unfavorable prognosis across various clinical subgroups (Fig. 3A, Fig. S1).

### Diagnostic and prognostic significance of PCDH8

As indicated by the ROC curve, PCDH8 effectively discerned thyroid tumors from healthy tissue (Fig. 3C, AUC = 0.747, 95% CI: 0.714–0.780), indicating its potential for identifying patients with THCA from healthy individuals. Favorable ROC results, with AUC values of approximately 0.7, were observed in specific subgroups (Fig. 3C). A nomogram and Forest diagram integrating clinical factors with PCDH8 expression were created, illustrating survival probabilities at 1, 3, and 5 years (Fig. 3D, E).

Cox regression analysis was conducted to evaluate the independent predictive value of PCDH8. Given the generally favorable prognosis and low mortality rate of THCA, we chose PFI as a prognostic measure. We identified several factors associated with PFI, including stage III and IV (HR = 2.615; *p* < 0.001), M1 staging (HR = 7.542, *p* < 0.001), T3 and T4 levels (HR = 2.507, *p* = 0.001), stage 4 (HR = 20.622, *p* < 0.001), and PCDH8 expression (HR = 1.959, *p* = 0.021). However, gender (*p* = 0.146), age (*p* = 0.092), and histological type (*p* = 0.246) exhibited no significant association with PFI (Table 2). In multivariate Cox regression, M1 staging (HR = 4.247, *p* = 0.005) and PCDH8 expression (HR = 2.419, *p* = 0.035) remained significantly correlated with PFI in patients with THCA (Table 2). Therefore, high expression of PCDH8 can independently predict a poorer prognosis of THCA.

**Table 2:** Univariate Cox regression and multivariate Cox regression analysis between PFI and clinical pathological factors in THCA patients

Characteristics	Total (*N*)	Univariate analysis		Multivariate analysis	
		Hazard ratio (95% CI)	*p* value	Hazard ratio (95% CI)	*p* value
Age	502				
< = 45	236	Reference			
> 45	266	1.618 (0.924–2.834)	0.092	1.362 (0.427–4.345)	0.601
Gender	502				
Female	367	Reference			
Male	135	1.529 (0.863–2.709)	0.146		
Pathologic stage	500				
Stage I & Stage II	333	Reference			
Stage III & Stage IV	167	2.615 (1.515–4.515)	< 0.001	2.641 (0.741–9.412)	0.134
Histological type	457				
Follicular	101	Reference			
Classical	356	1.666 (0.704–3.942)	0.246		
T stage	500				
T1&T2	307	Reference			
T3&T4	193	2.507 (1.434–4.385)	0.001	0.972 (0.367–2.575)	0.954
M stage	291				
M0	282	Reference			
M1	9	7.542 (2.860–19.891)	< 0.001	4.237 (1.529–11.741)	0.005
N stage	452				
N0	229	Reference			
N1	223	1.578 (0.885–2.814)	0.122		
PCDH8	502				
Low	251	Reference			
High	251	1.959 (1.106–3.469)	0.021	2.419 (1.062–5.510)	0.035

### Genetic modification of PCDH8 in THCA

**Fig. 4:**
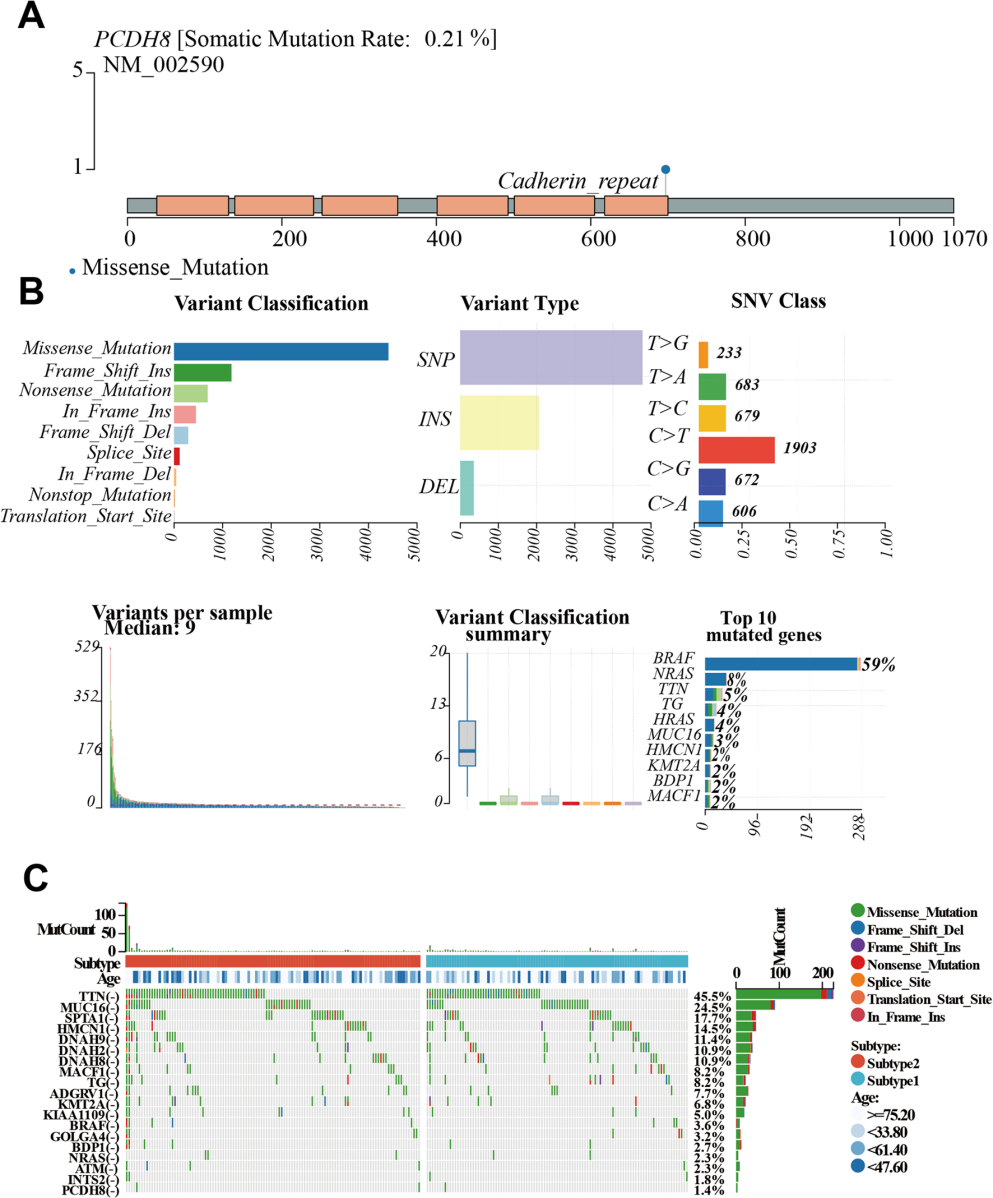
Investigation of the mutation landscape of PCDH8 (**A**) The location of PCDH8 in the genome. (**B**, **C**) The alteration landscape of PCDH8 in THCA

We mapped the genomic location of PCDH8 (Fig. 4A) to provide a comprehensive overview. Subsequently, we analyzed the overall gene mutation profile in patients with THCA, identifying nonsense mutations as the most frequent, with single nucleotide polymorphisms (SNPs) being the predominant type. C > T transitions were the most common nucleotide changes observed. On average, patients with THCA had a median of nine mutations. Notably, high mutation rates were observed in genes such as BRAF, NRAS, and TTN (Fig. 4B).

PCDH8 mutations were observed in 1.4% of patients with THCA, most of them being nonsense mutations (Fig. 4C). This suggests a potential PCDH8 impact on the development and progression of the disease in a small percentage of THCA cases. Understanding the genetic landscape of THCA, including the presence of PCDH8 mutations, is of critical importance. Further research is needed to elucidate the functional consequences of these mutations and their potential role in THCA pathogenesis.

### Correlation between PCDH8 expression and immune infiltration

**Fig. 5:**
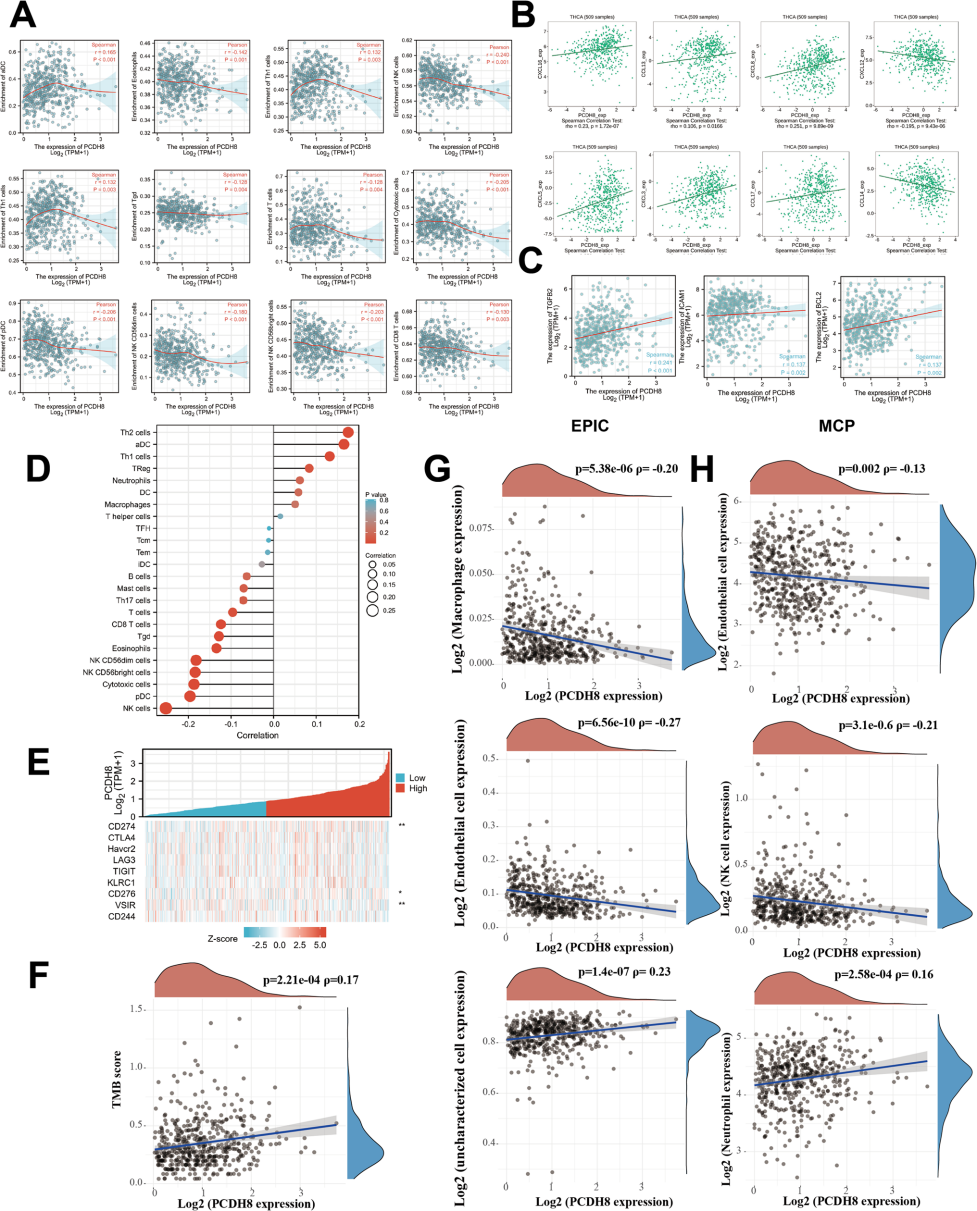
Immune infiltration analysis. (**A**) The association between 12 immune cell types and PCDH8 mRNA expression based on ssGSEA in THCA (single sample gene set enrichment analysis). (**B**) The correlation between PCDH8 expression and immune chemokines in THCA. (**C**) The association of PCDH8 expression with TGFB2, ICAM1 and BCL-2 of THCA patients. (**D**) The generally correlation between 24 immune cells and PCDH8 based on Estimate algorithm in THCA. (**E**) Heat map of single gene co-expression between PCDH8 and some vital immune checkpoints. (**F**) TMB (tumor mutation burden) of PCDH8 in THCA. (**G**, **H**) The association analysis of 3 immune cell types with PCDH8 mRNA expression based on EPI and MCP algorithm. MCP, a method based on marker gene sets to quantify tumor immune cells, fibroblasts, and epithelial cells. EPI, an immune algorithm that provides the relative components of cells. *p < 0.05; **p < 0.01; ***p < 0.001

The correlation between PCDH8 expression and immune cell infiltration was examined (Fig. 5D). PCDH8 expression was associated with the infiltration of various immune cells, including Th1, CD56^dim^, B, mast, T, activated dendritic (aDC), natural killer (NK), T helper, gamma-delta T (Tgd), CD56^bright^, dendritic (DC), plasmacytoid DC (pDC), cytotoxic, and CD8 + T cells as well as eosinophils. Specifically, PCDH8 expression exhibited a positive correlation with Th1 cells (*r* = 0.132, *p* = 0.003) and aDC (*r* = 0.165, *p* < 0.001), and negative correlations with T (*r* = -0.128, *p* = 0.004), NK (*r* = -0.240, *p* < 0.001), Tgd (*r* = -0.128, *p* = 0.004), CD56^bright^ (*r* = -0.203, *p* < 0.001), pDC (*r* = -0.206, *p* < 0.001), cytotoxic (*r* = -0.205, *p* < 0.001), CD8 + T, and B cells as well as eosinophils (*r* = -0.142, *p* = 0.001) (Fig. 5A). Furthermore, we utilized EPIC and MCP algorithms to evaluate immune infiltration levels (Fig. 5G, H), and calculated the TMB score (Fig. 5F). These findings further emphasize the significant role of PCDH8 in modulating immune infiltration within the tumor microenvironment.

To delve into the role of PCDH8 in the tumor immune process, we analyzed its association with immune checkpoints using the TIMER database and R package. As depicted in Fig. 5E, PCDH8 expression exhibited a significant positive correlation with key immune checkpoints, including B7-H3 (CD276) (*p* < 0.05), programmed cell death ligand 1 (PD-L1 CD274) (*p* < 0.01), and V-Set immunoregulatory receptor (VSIR; *p* < 0.01). Moreover, PCDH8 expression was positively associated with immunosuppression molecules such as B-cell lymphoma 2 (Bcl-2; *r* = 0.137, *p* = 0.002), intercellular adhesion molecule-1 (*r* = 0.137, *p* = 0.002), and tumor growth factor beta 2 (TGF-β2; *r* = 0.241, *p* < 0.001) (Fig. 5C). These findings suggest that PCDH8 may regulate immune infiltration and checkpoints. Additionally, we analyzed the relationship between PCDH8 and several chemokines, including chemokine (C–C) ligand (CCL)17, CCL14, CCL13, CXC chemokine ligand (CXCL)12, CXCL5, CXCL3, CXCL8, and CXCL16 (Fig. 5B). Previous studies have highlighted the significance of CXCL8 in thyroid cancer growth and progression (Bauerle et al. 2014; Fang et al. 2014; Visciano et al. 2015; Coperchini et al. 2016).

### GO and KEGG analyses

**Fig. 6:**
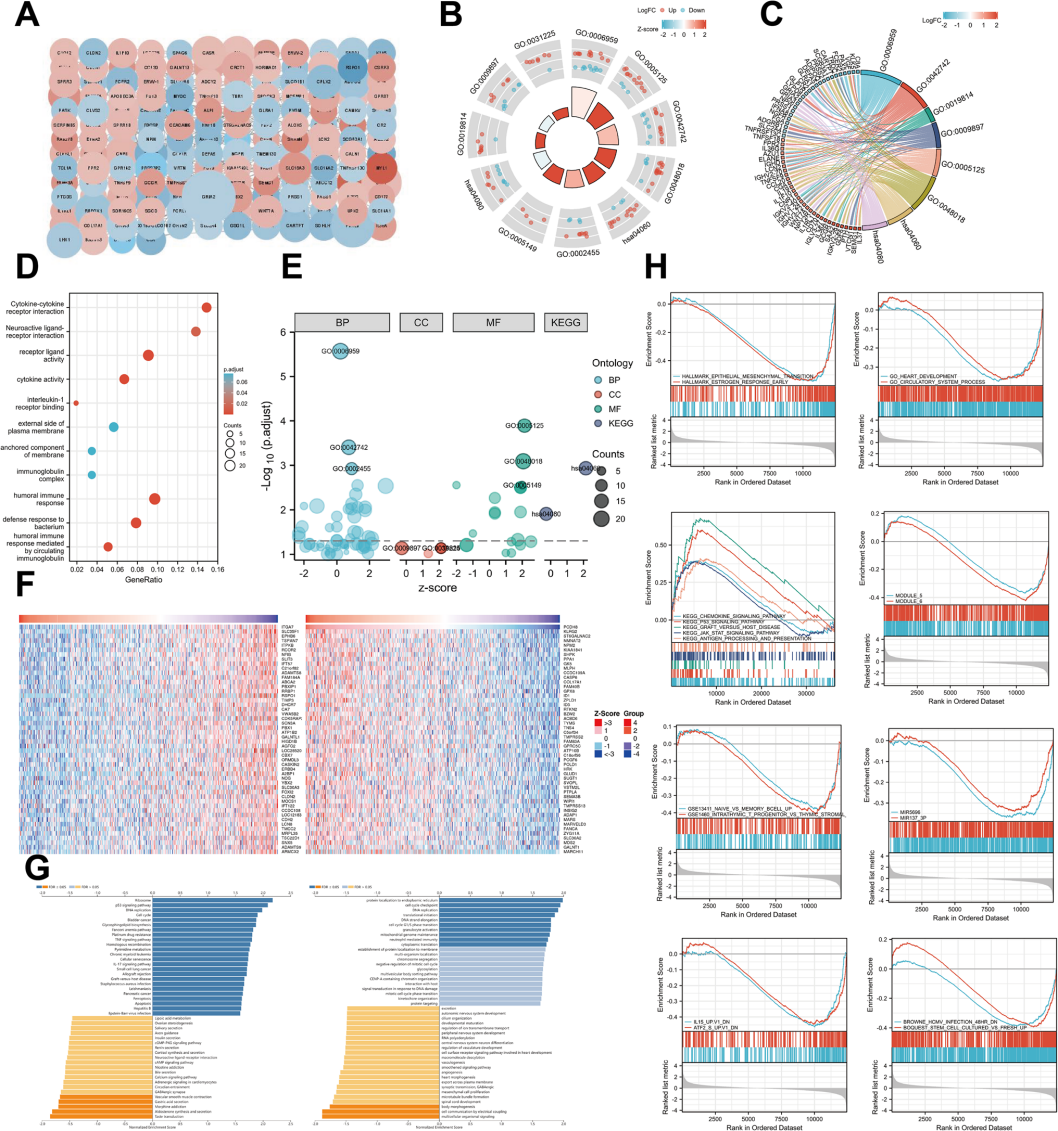
Functional enrichment analysis of top 415 DEGs between high and low PCDH8 expression groups and Co-expressed genes functional enrichment analysis of PCDH8 (LinkedOmics). (**A**) Protein–protein interaction network of top 415 DEGs. (**B**, **C**, **D**, **E**) GO and KEGG pathway analysis based on 415 related genes. (**F**) Heat maps indicate the top 50 genes positively and negatively correlated with PCDH8 in THCA. Red refers to positively correlated genes and green refers to negatively correlated genes. Statistical analysis was performed using Pearson’s test. (**G**) GO function and KEGG pathway analyses of PCDH8. (**H**) Gene set enrichment analysis. DEGs, differential expressed genes, GO, gene ontology, KEGG, Kyoto Encyclopedia of Genes and Genomes, FDR, False discovery rate

To investigate the biological functions of PCDH8 in THCA, we identified the top 415 associated DEGs based on |log2 Fold change|> 1 and *p* < 0.05 thresholds. Subsequently, we constructed a PPI network for these DEGs using the STRING database and Cytoscape software (Fig. 6A). The PPI network provided insights into potential interactions and functional relationships among these genes, revealing possible biological roles of PCDH8 in THCA.

We conducted a GO enrichment analysis. In the biological processes, genes were mainly enriched in humoral immune response, complement activation of the classical pathway, defense response to bacterium, leukocyte migration, and humoral immune response mediated by circulating immunoglobulin. In the cellular component, genes were enriched in immunoglobulin complex, circulating, external side of plasma membrane, and anchored component of membrane. The major molecular functions were cytokine activity, interleukin-1 receptor binding, receptor-ligand activity, and endopeptidase inhibitor activity (Fig. 6B, C, D, E). KEGG pathway analysis revealed enrichment in pathways such as cytokine-cytokine receptor interaction and neuroactive ligand-receptor interaction, closely related to tumor progression (Fig. 6C, D, and E).

### Identification of PCDH8-related signaling pathways using GSEA

Based on normalized enrichment scores (NES), we identified several pathways significantly associated with PCDH8 expression, such as cell cycle, p53 signaling, chemokine signaling, antigen processing and presentation, and graft versus host disease pathways (Fig. 6H). These findings suggest that PCDH8 may play a crucial role in the development and progression of THCA by participating in these metabolic pathways.

### LinkedOmics analysis

Using the LinkedOmics platform, we conducted a comprehensive analysis of PCDH-8-associated genes exhibiting differential expression in THCA. In total, 19,927 genes correlated with PCDH8. The top 50 genes with significant positive and negative correlations with PCDH8 are illustrated in Fig. 6F. Further analysis revealed that PCDH8 and its neighboring genes were primarily enriched in various biological processes, including cell cycle checkpoint, DNA replication, translational initiation, DNA strand elongation, neutrophil-mediated immunity, and chromosome segregation (Fig. 6G). Moreover, KEGG pathway analysis indicated their involvement in pathways such as ribosome, bladder cancer, small cell lung cancer, interleukin (IL-17) signaling pathway, leishmaniasis, and allograft rejection (Fig. 6G). These findings shed light on the specific mechanisms through which PCDH8 may contribute to the development and progression of THCA. Notably, enrichment in pathways associated with cell viability and proliferation was consistently observed across KEGG, GO, and GSEA analyses, strongly supporting the role of PCDH8 in THCA through its involvement in regulating cell viability and proliferation.

### PCDH8 kinase targets

**Fig. 7:**
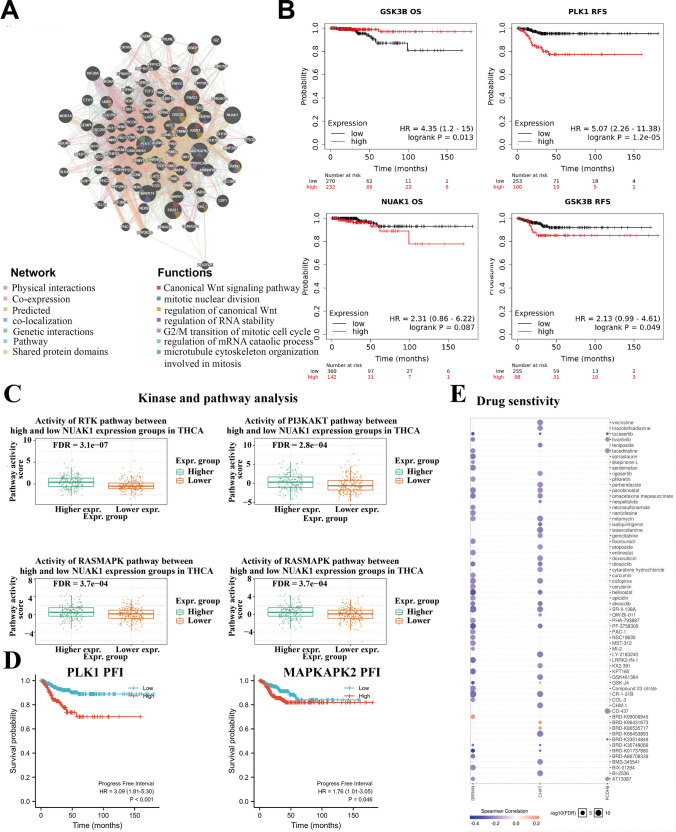
PPI networks of related kinase targets and their survival and clinical significance. (**A**) PPI networks of PCDH8 and most related kinase targets. (**B**, **D**) The association between kinase and prognosis. (**C**) The association between kinase and significant pathways. (**E**) Drug resistance analysis of the PCDH8

The LinkedOmics database was utilized to identify potential kinase targets of PCDH8 in THCA. The identified network included NUAK family SNF1-like kinase 1 (NUAK1), glycogen synthase kinase 3 beta, mitogen-activated protein kinase-activated protein kinase 2, and polo-like kinase 1 (PLK1). GeneMANIA was used to construct a PPI network and determine specific functions and potential regulatory mechanisms of these kinases and PCDH8 (Fig. 7A). The analysis revealed their involvement in the canonical wingless-type MMTV integration site family (Wnt) signaling pathway, mitotic nuclear division, regulation of mRNA catabolic process, regulation of RNA stability, G2/M transition of the mitotic cell cycle, and microtubule cytoskeleton organization involved in mitosis (Fig. 7A).

Additionally, we assessed the survival and clinical relevance of PCDH8-regulated kinases in THCA. Interestingly, all four kinases exhibited a significant association with poor prognosis in THCA (Fig. 7B, D). Specifically, NUAK1 was deeply involved in pathways such as RTK, PI3K-AKT, and RAS-MAPK, known to promote THCA development (Fig. 7C). These findings support the hypothesis that PCDH8 is involved and regulates THCA progression through downstream signaling pathways involving these kinases.

### Impact of PCDH8 on drug sensitivity

Using data from the Cancer Drug Sensitivity Genomics database, we investigated the relationship between PCDH8 expression and the IC50 values of various drugs. Our analysis revealed that reduced PCDH8 expression was significantly associated with the effectiveness of six molecules: AT13387, BRD-K33514849, CD-437, tacedinaline, tivantinib, and tozasertib (Fig. 7E). These findings suggest that PCDH8 is negatively regulated by these molecules, highlighting its potential as a promising therapeutic target for THCA. Further research and development of drugs targeting PCDH8 could improve treatment outcomes for patients with THCA.

### THCA samples and IHC

**Fig. 8:**
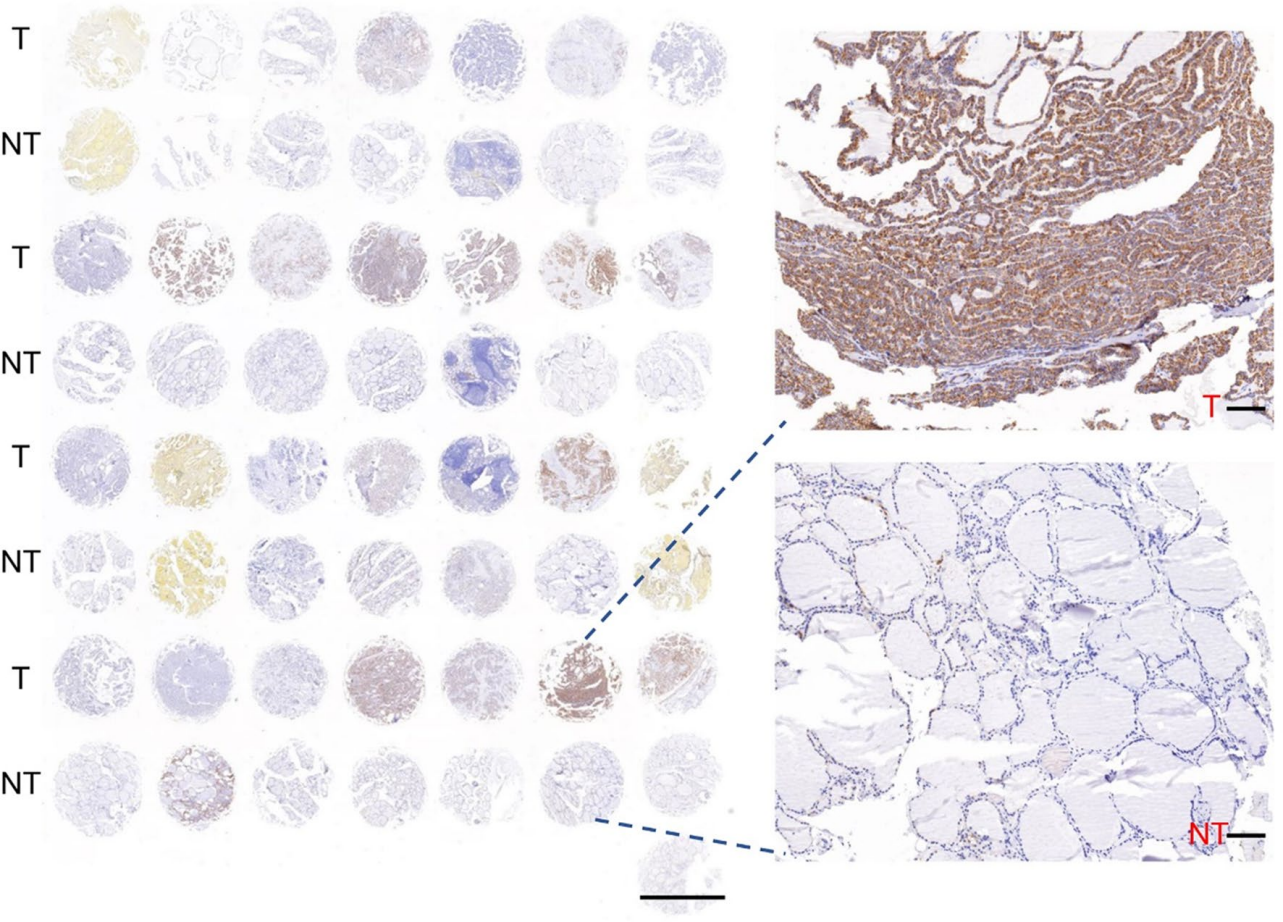
The expression of PCDH8 in THCA samples and matched adjacent non-tumorous specimens. The magnification of the left is × 0.4, and scale bars = 200 μm. The magnification of the right is × 10, and scale bars = 100 μm. T: human THCA samples; NT: adjacent non-tumorous specimens

We collected 98 human THCA samples and their adjacent non-tumor samples. Employing IHC staining, we demonstrated that PCDH8 expression was substantially higher in THCA samples compared to the adjacent non-tumorous specimens (Fig. 8, Fig. S2). These results are consistent with the other findings of the present analysis.

### MTT and colony formation assay

**Fig. 9:**
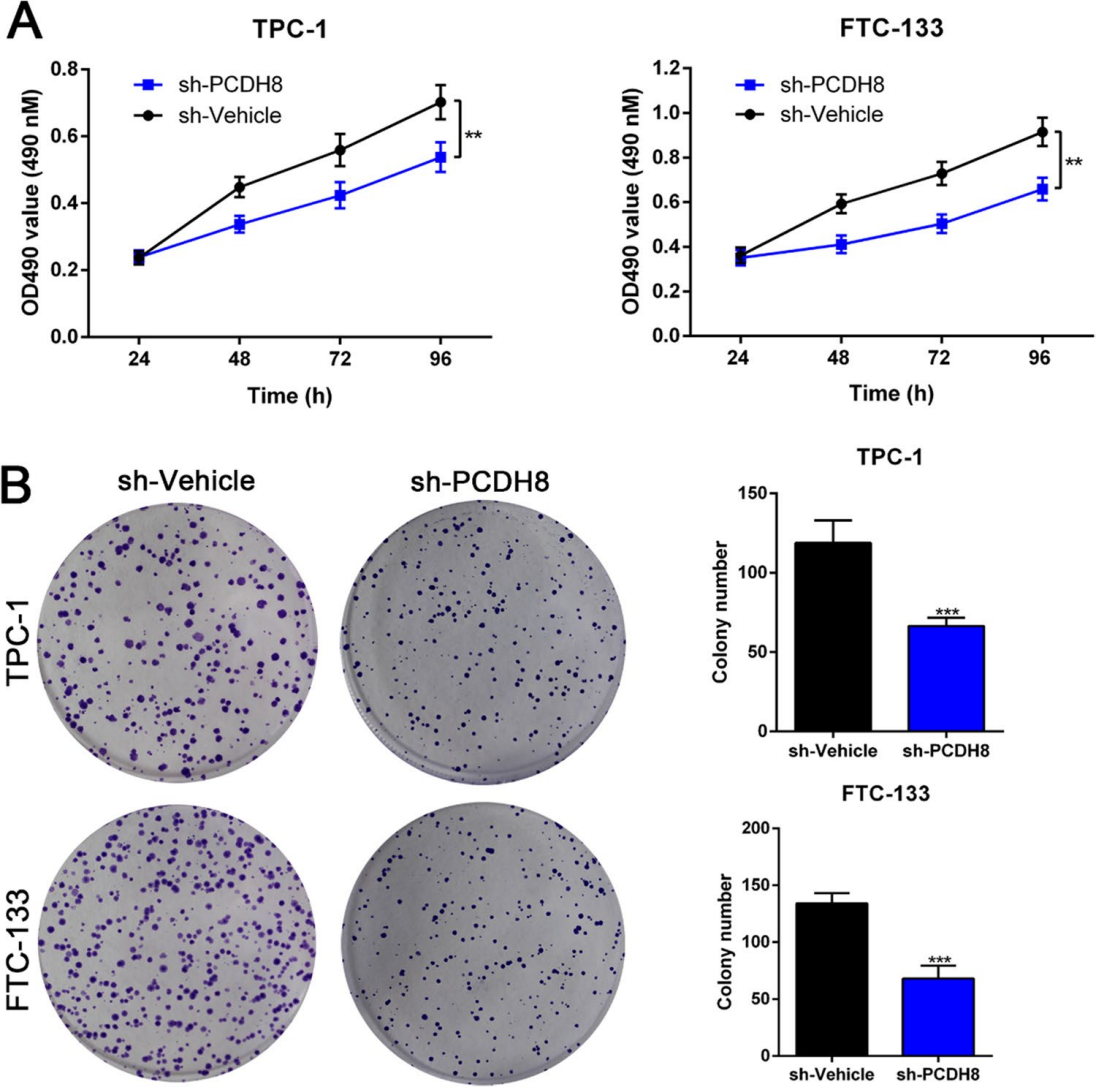
Depletion of PCDH8 obviously mitigated the proliferation of THCA cells. Knockdown of PCDH8 in THCA cells was achieved by transfection of PCDH8 shRNA, then the cell viability (**A**) and cell proliferation (**B**) were assessed by MTT and colony formation assay respectively. **p < 0.01, ***p < 0.001

Given the significant impact of PCDH8 on cell viability and proliferation pathways as indicated by KEGG, GO, and GSEA analyses, we performed MTT and colony formation assays to further explore its mechanism in THCA. Notably, the knockdown of PCDH8 in TPC-1 and FTC-133 cells substantially decreased cell viability and proliferation (Fig. S3, Fig. 9A, B). Thus, PCDH8 promotes tumor cell proliferation and viability in THCA, potentially being an important driver of THCA progression.

## Discussion

THCA, along with breast, lung, colorectal, and uterine cancers, is a frequently occurring malignant tumor with an increasing annual incidence (Siegel et al. 2020). Although patients with THCA typically have a favorable prognosis, those who experience recurrence or distant metastasis often face more adverse outcomes. Consequently, there is an essential need to identify patients with THCA early, a necessity for both researchers and clinicians. PCDHs play important roles in tumor cell adhesion, proliferation, apoptosis, and migration, potentially serving as prognostic biomarkers and therapeutic targets in THCA (Liu et al. 2019; Luo et al. 2019b; Vega-Benedetti et al. 2019; Fujiki et al. 2023). Exploring the role of PCDHs in THCA could provide valuable insights for effectively identifying and managing THCA.

PCDH8, a member of the PCDH family, encodes an integral membrane protein believed to contribute to cell adhesion. Its oncogenic role has been investigated in various types of cancer. In esophageal squamous cell carcinoma, PCDH8 is regulated by miR-200c and functions as a regulator of metastasis (Yu et al. 2020). In gastric cancer, PCDH8 promotes invasion and metastasis through enhanced interaction with extracellular matrix receptors, possibly through upregulation of laminin subunit gamma-2 (Lin et al. 2018). In glioma, PCDH8 might function as a biomarker for early detection and prognosis, its expression level being directly associated with glioma progression (Zong et al. 2017). However, the involvement of PCDH8 in THCA remains underexplored. Investigating its role in THCA could provide valuable insights into its potential as a biomarker or therapeutic target for this specific cancer type.

In this study, we conducted a comprehensive analysis of the role of PCDH8 in THCA. Pan-carcinoma analysis revealed a distinct difference in PCDH8 expression between tumor and noncancerous samples in THCA. This was validated through unpaired and paired t-tests using public databases. Additionally, staining results from the HPA database supported these findings, indicating that PCDH8 likely plays a crucial role in THCA development. These findings align with those of Lin et al., revealing that PCDH8 overexpression is associated with malignant features and poor prognoses (Lin et al. 2018). Additionally, we explored the association between PCDH8 expression and clinical factors in THCA, revealing significant correlations with age, primary neoplasm focus type (including multifocal and unifocal), PFI, and a history of thyroid gland disorders. Patients with multifocal THCA were more likely to experience recurrence or persistence compared to those with unifocal THCA. This is consistent with the results of Ting Zhang et al. indicating that multifocality is associated with tumor aggressiveness and plays a crucial role in tumorigenesis and cancer progression (Zhang et al. 2021). While the 2015 update of the ATA guidelines did not consider multifocality as a risk factor, these newer studies have shed light on its significance.

Cox regression analysis identified PCDH8 as an independent prognostic factor for predicting PFI in patients with THCA. The ROC curve analysis demonstrated high sensitivity and specificity, indicating the potential of PCDH8 as a diagnostic and prognostic biomarker in THCA. Further research on the underlying mechanisms and functional roles of PCDH8 in THCA could lead to novel therapeutic strategies for this disease. Tumor microenvironment immune cells and molecules play a crucial role in various cancers (Bai et al., 2023). Herein, we observed a negative correlation between PCDH8 expression and immune infiltration. The degree of immune cell infiltration strongly impacts patient prognosis in solid tumors (Ino et al. 2013; Schneider et al. 2018). Our findings revealed significant associations between PCDH8 and various immune cells, molecules, and checkpoints, including Th1 cells, aDC,, T cells, eosinophils, NK cells, Tgd cells, CD56 bright cells, pDC, cytotoxic cells,, cytotoxic T-lymphocyte-associated protein 4, Bcl-2, tumor growth factor beta 2 (TGF-β, PD-L1, CD276, VSIR and some chemokines. Thus, the PCDH8 gene affects immune cells and related molecules in the tumor microenvironment, and its overexpression is correlated with poorer prognosis in THCA. Specifically, the positive correlation of PCDH8 with negative immune checkpoints (CTLA-4, PD-L1, CD276) and negative regulator cytokines such as TGF-β, known to reduce immune infiltration (Krummel and Allison 1995; Keir et al. 2008), suggests a potential role in immune escape. This mechanism contributes to THCA progression. The expression of PD-L1 in thyroid cancer has been extensively studied in prior research (Ulisse et al. 2019). In summary, PCDH8 may be involved in thyroid cancer by inhibiting immune infiltration and promoting immune escape.

Enrichment analysis between high and low PCDH8 expression groups was carried out to uncover its potential mechanisms in THCA. GO analysis revealed significant enrichment in pathways related to humoral immune response, complement activation of classical pathway, defense response to bacteria, leukocyte migration, humoral immune response mediated by circulating immunoglobulin, immunoglobulin complex, cytokine activity, and interleukin-1 receptor binding. These pathways align with the role of PCDH8 in the tumor microenvironment and immune response.

Using GSEA, we identified several key pathways associated with PCDH8 phenotype, including cell cycle, cytokine-cytokine receptor interactions, p53 signaling, chemokine signaling, Janus kinase-signal transducer and activator of transcription signaling pathway, antigen processing and presentation, and graft versus host disease pathways. These pathways indicate the potential involvement of PCDH8 in cell cycle regulation, immune signaling, and cancer-related pathways.

Additional analysis using the LinkedOmics database revealed additional pathways and kinase targets associated with PCDH8. These pathways included the canonical Wnt signaling pathway, mitotic nuclear division, regulation of RNA stability, G2/M transition of mitotic cell cycle, and regulation of mRNA catabolic process, providing insights into downstream regulatory mechanisms of PCDH8 in cancer progression. Consistently, these findings emphasize the significance of cell cycle regulation, nucleic acid stabilization, and metabolism in cancer. Dysregulation of the cell cycle machinery and checkpoints contribute to aberrant cell divisions and genomic instability, which are major hallmarks of cancer. We hypothesize that PCDH8 contributes to THCA by regulating cell viability and proliferation.

To validate our findings, we performed experimental validation including THCA sample collection, IHC, cell culture, shRNA transfection, MTT, and colony formation experiments. IHC revealed significantly elevated PCDH8 expression in THCA tumor tissue compared to paired paraneoplastic tissue. Knockdown of PCDH8 in TTC-1 and FTC-113 cell lines significantly decreased cell proliferation and viability, as revealed by MTT and colony formation assays. These experimental results support our analysis, indicating that PCDH8 is involved in THCA tumorigenesis by promoting cell proliferation and viability and suppressing immune infiltration.

PCDH8 expression was regulated by drugs, including AT13387, BRD-K33514849, CD-437, tacedinaline, tivantinib, and tozasertib. These results support the use of PCDH8 as a potential biomarker and therapeutic drug target for improving THCA prognosis. Nevertheless, further in vivo validation is needed.

In conclusion, PCDH8 overexpression served as an independent prognostic predictor for patients with THCA, possessing high diagnostic efficacy. Moreover, its elevated expression in THCA was associated with biological processes and pathways such as cell viability and proliferation. Our study revealed PCDH8 as a significant prognostic biomarker and promoter of tumorigenesis in THCA.

### Supplementary Information

Below is the link to the electronic supplementary material.

Supplementary file1 (ZIP 6288 KB)

Supplementary file2 (ZIP 103870 KB)

## Data Availability

Details about databases, web tools and R packages can be found in the “Supplement 2 Details and links of the online tools and database”. TCGA(https://www.cancer.gov/about-nci/organization/ccg/research/structural-genomics/tcga/studied-cancers/thyroid), TIMER 2.0 online tool repository (http://timer.cistrome.org), GEPIA 2.0 online tool (http://gepia2.cancer-pku.cn), TISIDB database (http://cis.hku.hk/TISIDB/index.php). STRING website (https://stringdb.org/), Linkomics(http://www.linkedomics.org/), GSCALite(http://bioinfo.life.hust.edu.cn/web/GSCALite/). Gene MANIA(www.genemania.org), R software (https://bioconductor.org/biocLite.R), Enrichplot(https://bioconductor.org/packages/release/bioc/html/enrichplot.html). clusterProfiler(https://bioconductor.org/packages/release/bioc/html/clusterProfiler.html). ggplot2(https://cloud.r-project.org/web/packages/ggplot2/).

## Abbreviations

PCDH8: Protocadherin 8
THCA: Thyroid cancer
EMT: Epithelial-mesenchymal transition
IHC: Immunohistochemical
GSCA: Gene Set Cancer Analysis
DEGs: Differentially expressed genes
HPA: Human Protein Atlas
ROC: Receiver operating characteristic
AUC: Area under the curve
HR: Hazard ratios
PFI: Progression-free interval
GO: Gene Ontology
KEGG: Kyoto Encyclopedia of Genes and Genomes
STRING: Search tool for recurring instances of neighbouring genes
GSEA: Gene set enrichment analysis
TMB: Tumor mutation burden
CTRP: Cancer Therapeutics Response Portal
MHC: Major histocompatibility complex
PPI: Protein-protein interaction
TG: Thyroglobulin
