# Supplementary figures and images for: PCDH8 is a novel prognostic biomarker in thyroid cancer and promotes cell proliferation and viability
Source: Funct Integr Genomics. 2024 Feb 17;24(2):35. doi: 10.1007/s10142-024-01312-3 (PMC10874333; doi:10.1007/s10142-024-01312-3)

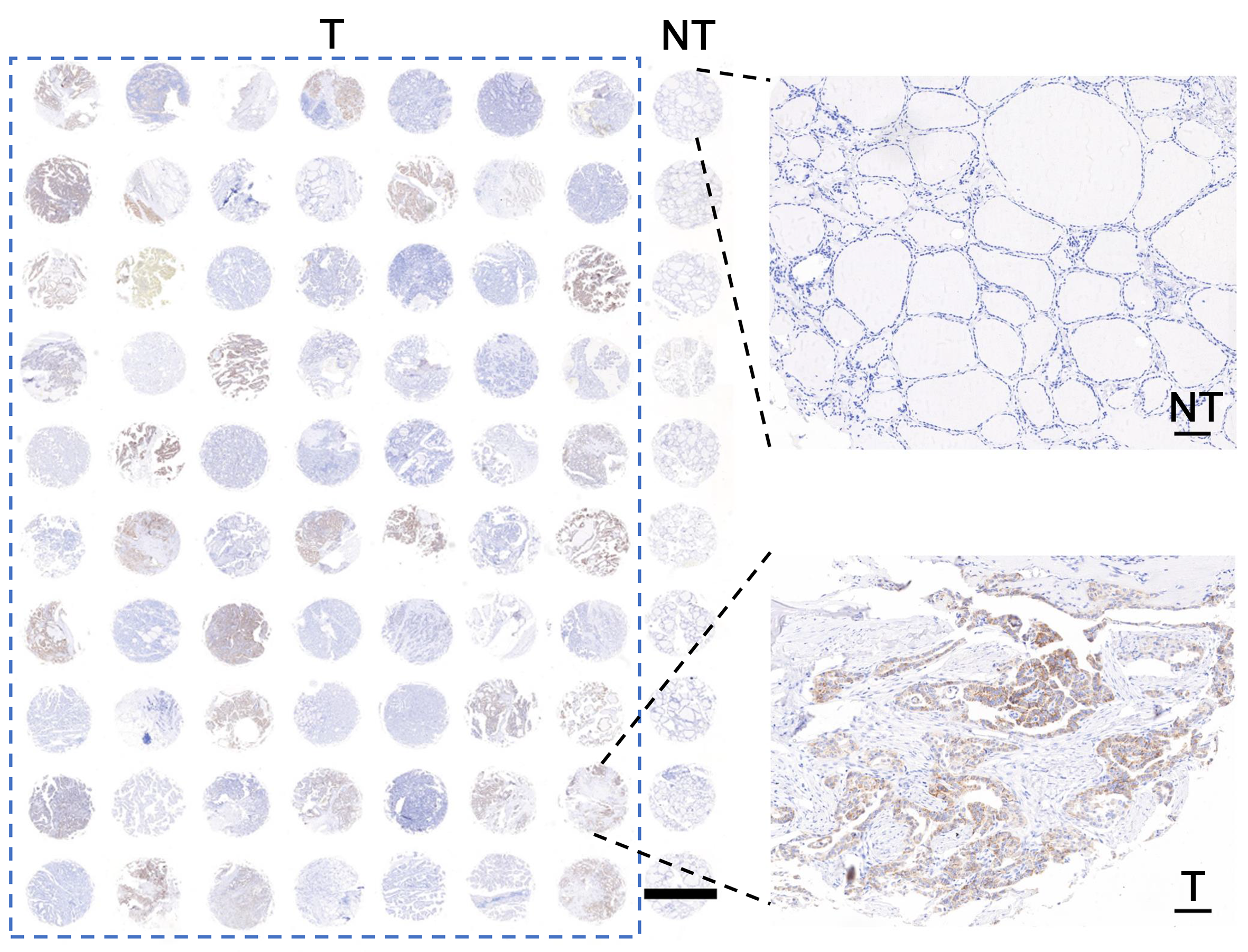

Supplement: Supplementary file 1 — Supplementary file1 (ZIP 6288 KB) [file 10142_2024_1312_MOESM1_ESM.zip › FIG S2.tif]

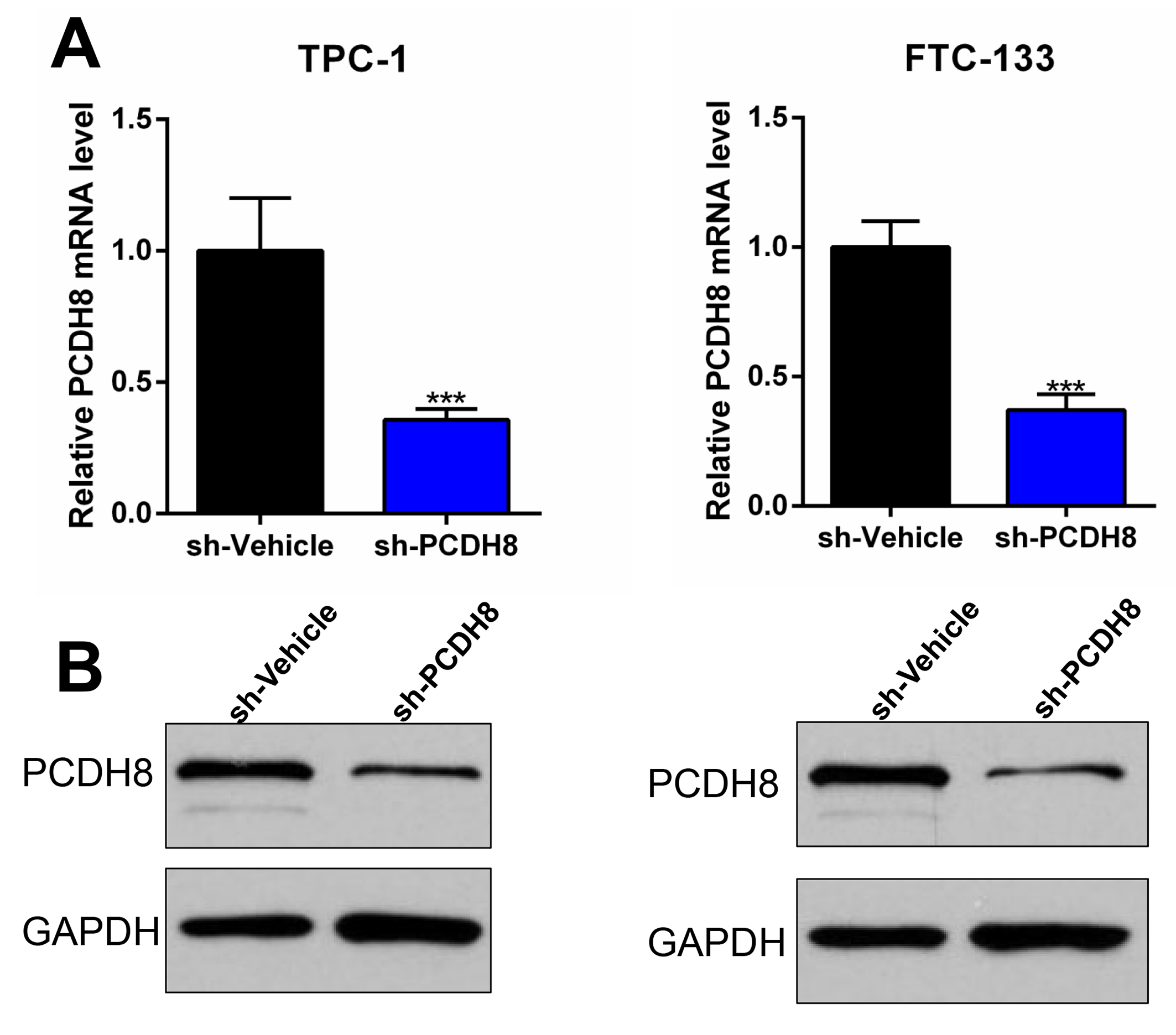

Supplement: Supplementary file 1 — Supplementary file1 (ZIP 6288 KB) [file 10142_2024_1312_MOESM1_ESM.zip › FIG S3.tif]

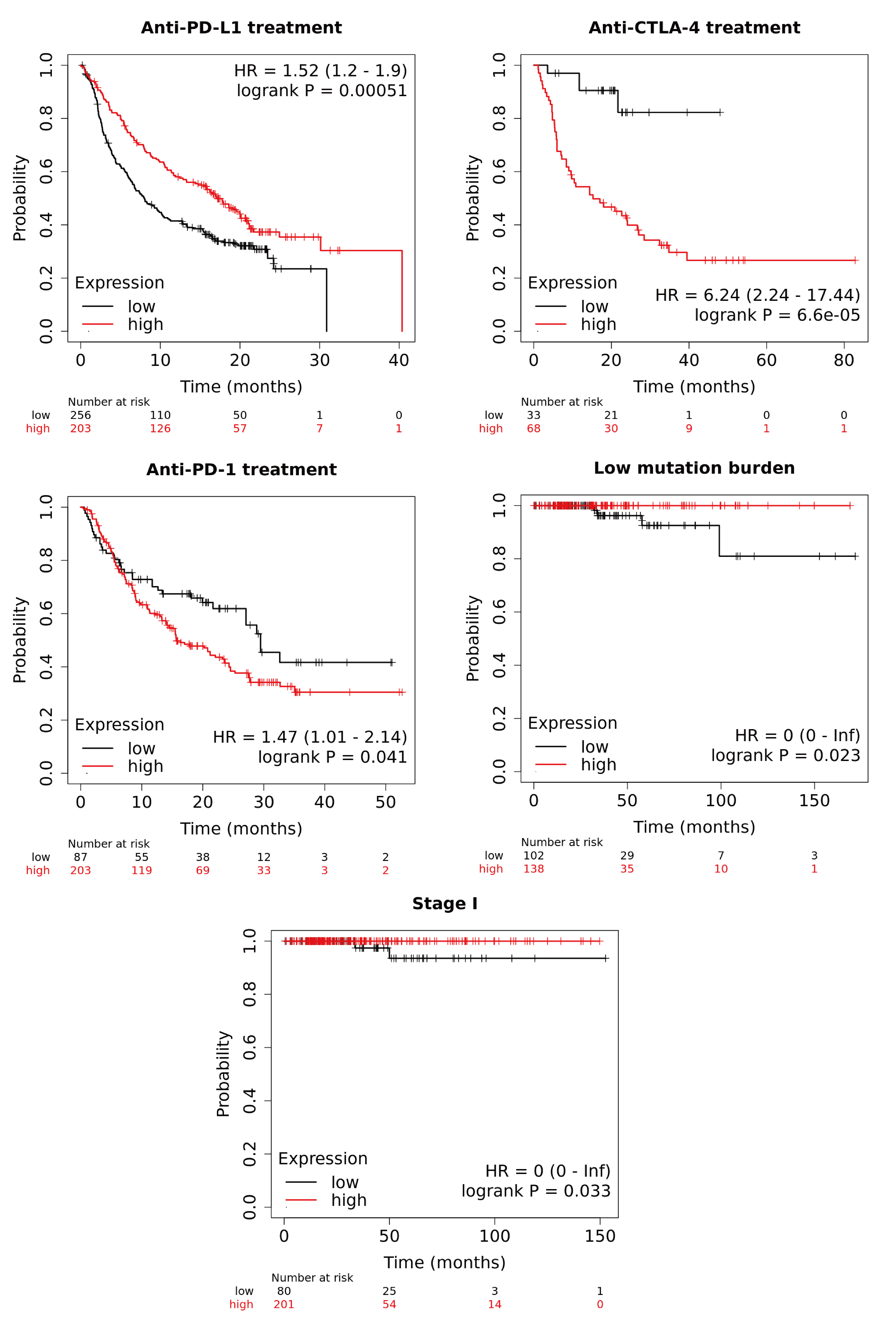

Supplement: Supplementary file 1 — Supplementary file1 (ZIP 6288 KB) [file 10142_2024_1312_MOESM1_ESM.zip › FIG S1.tif]
