# Supplementary material for: PCDH8 is a novel prognostic biomarker in thyroid cancer and promotes cell proliferation and viability
Source: Funct Integr Genomics. 2024 Feb 17;24(2):35. doi: 10.1007/s10142-024-01312-3 (PMC10874333; doi:10.1007/s10142-024-01312-3)
Supplement: Supplementary file 2 — Supplementary file2 (ZIP 103870 KB) [file 10142_2024_1312_MOESM2_ESM.zip › Supplementary materials/Supplement 2 Details and links of the online tools and database.docx]

**Details and links of the online tools and databases used in the article are listed below:**

1. TCGA:

**<https://portal.gdc.cancer.gov/>**

The Cancer Genome Atlas (TCGA), a landmark cancer genomics program, molecularly characterized over 20,000 primary cancer and matched normal samples spanning 33 cancer types. This joint effort between NCI and the National Human Genome Research Institute began in 2006, bringing together researchers from diverse disciplines and multiple institutions.

We used transcriptomic and clinical data from the TCGA-THCA project in TCGA, with transcriptomic data in FPKM format.

1. GSCA:

**<https://bioconductor.org/packages/release/bioc/html/maftools.html>**

Gene Set Cancer Analysis (GSCA) is an integrated platform for genomic, pharmacogenomic, and immunogenomic gene set cancer analysis.

1. Enrichplot package:

**<https://bioconductor.org/packages/release/bioc/html/enrichplot.html>**

The 'enrichplot' package implements several visualization methods for interpreting functional enrichment results obtained from ORA or GSEA analysis. It is mainly designed to work with the 'clusterProfiler' package suite. All the visualization methods are developed based on 'ggplot2' graphics.

1. clusterProfiler package:

**<https://bioconductor.org/packages/release/bioc/html/clusterProfiler.html>**

This package supports functional characteristics of both coding and non-coding genomics data for thousands of species with up-to-date gene annotation. It provides a univeral interface for gene functional annotation from a variety of sources and thus can be applied in diverse scenarios. It provides a tidy interface to access, manipulate, and visualize enrichment results to help users achieve efficient data interpretation. Datasets obtained from multiple treatments and time points can be analyzed and compared in a single run, easily revealing functional consensus and differences among distinct conditions.

1. ggplot2 package:

**<https://cran.r-project.org/web/packages/ggplot2/index.html>**

A system for 'declaratively' creating graphics, based on "The Grammar of Graphics". You provide the data, tell 'ggplot2' how to map variables to aesthetics, what graphical primitives to use, and it takes care of the details.

1. Limma package:

**<https://bioconductor.org/packages/release/bioc/html/limma.html>**

Data analysis, linear models and differential expression for microarray data.

1. pROC package:

**<https://cran.r-project.org/web/packages/pROC/index.html>**

Tools for visualizing, smoothing and comparing receiver operating characteristic (ROC curves). (Partial) area under the curve (AUC) can be compared with statistical tests based on U-statistics or bootstrap. Confidence intervals can be computed for (p)AUC or ROC curves.

1. Survival package:

**<https://cran.r-project.org/web/packages/survival/>**

Contains the core survival analysis routines, including definition of Surv objects, Kaplan-Meier and Aalen-Johansen (multi-state) curves, Cox models, and parametric accelerated failure time models.

1. Maftools package:

**<https://bioconductor.org/packages/release/bioc/html/maftools.html>**

Analyze and visualize Mutation Annotation Format (MAF) files from large scale sequencing studies. This package provides various functions to perform most commonly used analyses in cancer genomics and to create feature rich customizable visualzations with minimal effort.

1. GeneMANIA:

**<https://genemania.org/>**

GeneMANIA finds other genes that are related to a set of input genes, using a very large set of functional association data. Association data include protein and genetic interactions, pathways, co-expression, co-localization and protein domain similarity.

1. Linkomics:

**<https://linkedomics.org/>**

LinkedOmics is publicly available portal that includes multi-omics data from all 32 TCGA Cancer types and 10 Clinical Proteomics Tumor Analysis Consortium (CPTAC) cancer cohorts.The web application has three analytical modules: LinkFinder, LinkInterpreter and LinkCompare. LinkFinder allows users to search for attributes that are associated with a query attribute, such as mRNA or protein expression signatures of genomic alterations, candidate biomarkers of clinical attributes, and candidate target genes of transcriptional factors, microRNAs, or protein kinases.

1. STRING:

**https://cn.string-db.org/**

STRING is a database of known and predicted protein-protein interactions. The interactions include direct (physical) and indirect (functional) associations; they stem from computational prediction, from knowledge transfer between organisms, and from interactions aggregated from other (primary) databases.

1. TISIDB:

**<http://cis.hku.hk/TISIDB/index.php>**

TISIDB is a web portal for tumor and immune system interaction, which integrates multiple heterogeneous data types.

1. GEPIA

**<http://gepia.cancer-pku.cn/>**

GEPIA is a newly developed interactive web server for analyzing the RNA sequencing expression data of 9,736 tumors and 8,587 normal samples from the TCGA and the GTEx projects, using a standard processing pipeline.GEPIA provides customizable functions such as tumor/normal differential expression analysis, profiling according to cancer types or pathological stages, patient survival analysis, similar gene detection, correlation analysis and dimensionality reduction analysis.

1. HPA:

**<https://www.proteinatlas.org/>**

The Human Protein Atlas is a Swedish-based program initiated in 2003 with the aim to map all the human proteins in cells, tissues, and organs using an integration of various omics technologies, including antibody-based imaging, mass spectrometry-based proteomics, transcriptomics, and systems biology. All the data in the knowledge resource is open access to allow scientists both in academia and industry to freely access the data for exploration of the human proteome.
