# Supplementary material for: PCDH8 is a novel prognostic biomarker in thyroid cancer and promotes cell proliferation and viability
Source: Funct Integr Genomics. 2024 Feb 17;24(2):35. doi: 10.1007/s10142-024-01312-3 (PMC10874333; doi:10.1007/s10142-024-01312-3)
Supplement: Supplementary file 2 — Supplementary file2 (ZIP 103870 KB) [file 10142_2024_1312_MOESM2_ESM.zip › Supplementary materials/Supplement 6 Editoral certificates.pdf]

Bullet Edits

EDITORIAL CERTIFICATE

This document certifies that the paper listed below was edited and proofread for proper English language, grammar, punctuation, spelling, and overall style by one or more than one highly qualified native speakers at Bullet Edits. All of the suggested amendments were tracked with the Microsoft Word "Track Changes" feature. Therefore, the author had the option to reject or accept each change individually.

Manuscript Title:

PCDH8 is a novel prognostic biomarker in thyroid cancer and promotes cell proliferation and viability

Anti-counterfeiting Code:

293684bfd5b5d1fa9f4c51eea1c92c56

Date Issued:

2024-02-02 22:34:19

Bullet Edits is a registered company headquartered in the UK with a global presence. We offer a range of editing, proofreading services to authors. Our Ph.D. editors are all native English speakers from the USA and UK. Authors who work with Bullet Edits are guaranteed excellent language quality and timely delivery.

BULLET EDITS LIMITED COMPANY NO. 13345261 SEAL ★ ENGLAND AND WALES ★

Address: BULLET EDITS LIMITED, 85 Great Portland Street, London,UK
Tel: UK. (+44)20457 70286 / US.(+1)312-313-9179
Web: www.bulletedits.cn/Email: info@bulletedits.cn
VAT: GB 378 9316 43
